# Parental Life-Limiting Illness: What Do We Tell the Children?

**DOI:** 10.3390/healthcare7010047

**Published:** 2019-03-20

**Authors:** Rachel Fearnley, Jason W. Boland

**Affiliations:** Wolfson Palliative Care Research Center, Hull York Medical School, University of Hull, Hull HU6 7RX, UK; Jason.boland@hyms.ac.uk

**Keywords:** life-limiting illness, end of life, death, communication, information, children, parents, health care professionals

## Abstract

Being diagnosed with and having a life-limiting illness is a stressful experience which is compounded when the patient has dependent children. An important aspect of the patient’s psychosocial care should include recognition that their children are also likely to experience severe stress because of the illness. However, children’s needs are often overlooked during the illness. These needs include information about the illness. Health care professionals have a significant role in supporting patients to communicate with their children. This study aims to increase our understanding of children’s experiences when a parent has a life-limiting illness by exploring bereaved children’s experiences of the support they received when their parent had a life-limiting illness, and professionals’ perspectives of the support offered to children. 7 children (aged between 9 and 24), and 16 health care professionals were interviewed about communication during parental illness. Children report needing open, clear and age appropriate conversations with parents and health care professionals to help them begin to obtain some meaning from the situation. The importance of communication is discussed, with particular reference to the role health care professionals have in supporting these conversations.

## 1. Introduction

Children are exposed to significant levels of psychosocial stress when a parent is diagnosed with a life-limiting illness [1]. Their parent’s illness becomes a feature of daily life which requires pragmatic and psychological adaptation [2]. All that was previously known within the family takes on a different perspective as their parent’s illness progresses [2]. Every aspect of family life (e.g., routines) is disrupted and the predictable becomes unpredictable [2,3]. The illness and treatments have a significant impact on the lives of children, families and parents [4].

Parents are not only required to face the challenges of coping with the illness and its treatment, but additionally are exposed to the challenges of meeting their children’s needs including talking to them about the illness [1,5]. The illness may impact on the efficacy of their parenting, which can be severely diminished, and their relationship with their children destabilised [6]. Parents may be emotionally or physically unavailable for their children because of the illness [2], however, generally they work hard to maintain normalcy for them [7]. When parents have cancer, the physical and psychological burdens may limit their awareness and capacity to respond to their children’s needs [4].

One of the most difficult aspects for parents is having conversations with their children about the diagnosis, treatments and implications of the life-limiting diagnosis [8]. Interactions between the ill parent and their children, and the well parent and the children are likely to be different during the illness and will change over time. Evidence suggests that it is generally optimal to tell children about their parent’s illness so that they can begin to make meaning from what is happening [1,8,9,10]. Ideally these conversations need to be in ‘small chunks’, delivering manageable, age-appropriate amounts of information which become more detailed as the illness progresses [8]. However, it should also be recognized that children will often seek out information from their parents and other significant adults, for example relatives, when they become aware that their parent is ill.

There are many reasons why parents choose not to talk to their children about their life-limiting illness. They may have the misconception that their children are too young to understand and therefore do not need to be included in what is happening [8,10]. However, children as young as three have some understanding of what is happening in families when a parent has a life-limiting illness [10]. The reluctance to talk to children about the illness is sometimes because parents do not know what to say, and do not know how to start the conversation [1]. In these situations, support from health care professionals (HCPs) could be invaluable in facilitating conversations between parents and their children. Professional intervention may help patients prepare to tell their children, while also considering the ways children at different developmental stages may react [5]. However, there often remains an unspoken assumption, from the HCP’s perspective, that the patient’s needs are paramount at the expense of other family members’ needs [11]. This is true in many ways; however, a wider perspective should be adopted where children are recognized as also being in need of help and support during this potentially difficult period. The present study adds to the literature through the inclusion of children as primary participants in the research, through which their voices can be heard.

Health care professionals have reported that working with families is one of the most difficult aspects of working with people who have a life-limiting illness [12]. Moreover, when clinicians do not perceive that it is their role to deal with psychosocial concerns, they are less likely to encourage any communication with patients about their children [13]. A systematic review showed that there is a need for additional training and support for HCPs to help manage complex situations [14]. Communication, between HCPs and caregivers of people with advanced cancer, can be difficult but is needed [15]. To do this, HCPs must develop and continue to improve their communication about end-of-life issues in patients with life-limiting illness [15,16,17]. This need for effective communication is required if HCPs are to accurately understand and help manage the holistic needs of patients [18].

There is a paucity of research exploring children’s experiences when a parent has a life limiting illness [19,20]. Children’s accounts of how they interpret and understand the issues of living with an ill parent are fundamental to addressing this gap in the literature. In this study, children’s and HCP’s experiences of living and working with parental life-limiting illness are explored. We will discuss the importance of communication in ensuring that the needs of children, whose parents have a life-limiting illness, are recognized and supported. This will be integrated with a discussion exploring the role of HCPs in facilitating these conversations. Throughout the article, we will use ‘parent’ to refer to the patient who has responsibility for children. This includes biological parents, step-parents or other primary caregivers. The well parent can also have a significant role in supporting their children during the illness (and beyond), and this role is likely to be different from the ill parent’s role. ‘Children’ will be used to refer to any child or young person under the age of 18 as defined by the Children Act (1989). Two children were over 18 at the time of the interviews but were under 18 when their parent was ill and died. In addition, although the emphasis is on HCPs, there is also recognition of the role social care professionals have in working with children whose parents have a life-limiting illness.

The study aims to increase our understanding of children’s experiences when a parent has a life-limiting illness. Therefore, it examines children’s and professional’s narratives about their experiences of living with and working with parental life-limiting illness. This includes how information about the illness is communicated and the quality and quantity of support that is available during this period. The study has the following objectives:

▪ To explore bereaved children’s experiences of the support they received when their parent had life-limiting illness.
▪ To investigate with professionals the support offered to children.

## 2. Materials and Methods

This is a qualitative study that examines emotional and social factors in children whose parents have a life-limiting illness. The epistemology for the research was concerned with children’s experiences of living with a parent who had a life-limiting illness. The children’s stories are their account of what happened to them and their interpretation of events. Consequently, this research is not concerned with discovering truths about their lives, but rather how they make sense of what happened, and how they interpreted their experiences within this. Therefore, their narratives are memories of the events that happened, and which are subject to change and reinterpretation [21]. They represent a snapshot abstracted from the present [22]. It was important to not only capture the children’s stories and experiences but also to engage with professionals, involved in end of life care, to hear their perspectives. This then provided a different set of narratives which offered insight into children’s experiences from adults’ viewpoints.

Semi-structured interviews with children bereaved of a parent and professionals working in different settings that provide palliative care, including nurses, social workers and bereavement counsellors, were undertaken. The focus was on participant experiences of living and working with parental life-limiting illness.

### 2.1. Context of the Study

The aim of the study was to develop an understanding of children’s experiences of living with a parent who had life-limiting illness.

### 2.2. Recruitment

Recruitment of participants occurred through purposive sampling [23], thus allowing a range of perspectives to be explored which were representative of the planned research population, including children bereaved of a parent, and professionals working within palliative care and allied fields. Requests were made in writing to four hospices and specialist palliative care centers for their support in identifying potential HCPs and children to participate in the research. From these initial contacts other health and social care professionals, along with different service providers, for example charities supporting specific life-limiting conditions, were identified. The initial contacts with the hospices and specialist palliative care centers were important in assisting with the identification and recruitment of participants. In addition, children and young people, who had experienced parental bereavement, were identified as potential participants by the professionals who had consented to participate in the research. The inclusion criteria for the research were that the parent’s death was as a result of life-limiting illness and not through sudden or accidental death and that there had been professional involvement with regards to the children during the parental illness. Children younger than five (at the time of the interviews) were excluded however there were no exclusions regarding the time since the death of the parent. Decisions regarding the inclusion/exclusion criteria were made by the first author in conjunction with their Director of Studies, who was responsible for overseeing the research.

### 2.3. Participants

The experiences of seven children bereaved of a parent were included in the research. The focus of the research was on their experiences prior to the death of their parent. Five participants were females aged between six years and twenty-three years and two were males aged between nine and twenty-one years (Table 1). The sixteen professionals involved supported children whose parents had a life-limiting illness; their roles included nurses (n = 4), social workers (n = 5) and other support workers (n = 7). This range of health and social care practitioners was included in the research because of their experience and knowledge of working in this area. They all worked within palliative care or with children and families who had been bereaved. No new potential participants were approached when saturation of the data had been achieved, that is no new themes or information was emerging [24]. Potential participants were provided with written information about the research. These were carefully developed for the children and reflected different ages and stages of development. The parents were also provided with an information sheet and were asked to give written consent for their children to participate in the research. Children over 16 were asked to give consent while younger participants were asked to assent to being involved. Throughout the fieldwork, children were assured that they had choice about participation and that even if their parents consented to them taking part they could chose not to. Furthermore they were reminded that they could withdraw from the research at any time.

### 2.4. Data Collection

Semi-structured interviews were conducted individually with children and professionals. They were undertaken by R.F. For the children, these were all undertaken in their own homes. The under 18-year olds (at the time of the interviews) were asked whether they would like their parent or another adult to be with them during the interview. The younger children (Emma and Sonny) chose to be interviewed with their parents present. It felt important and appropriate that their parents were alongside them during the interview for support and encouragement. It could be deemed that this had a negative effect on the data, as it could be suggested that the children were reluctant to share some memories for fear of upsetting their parent. However, it could also be noted as a positive as the children may have felt more confident because they had their parent alongside them.

The interviews with professionals were undertaken at their place of work. Interviews with professionals ranged from forty minutes to two hours. ‘Hannah’ was interviewed twice, while the other professionals were all interviewed once. A similar interview schedule was followed as for the interviews with the children.

An interview schedule was developed which included key prompts to encourage conversations about experiences of living or working with parents with life-limiting illness. The prompts included asking about memories of:

- Conversations about the illness.
- Timings of conversations.
- Who initiated conversations?
- The extent of available support.
- The children’s wishes and feelings about the support.

This enabled the interviews to develop so that conversations with a purpose were being held [25]. This approach was consistent with the underpinning values of the research where the voice of the participant was paramount and where their memories and narratives were central [26].

An interview protocol was developed which was followed during each interview. This included re-confirming informed consent, reiterating the reasons for the interview, explaining about confidentiality and anonymity and reassuring the participant that they could terminate the interview at any point. In addition, the interviews with children were appropriately adapted to take their age and level of understanding into consideration. The researcher was an experienced practitioner who had worked directly with children for many years. Because of the sensitive nature of the research, it was important that throughout the process there was vigilance to ensure that the participant was not experiencing distress because of their involvement. The length of interviews ranged from forty minutes to two hours. At the end of each interview an endings protocol was followed which included thanking the participant, checking their emotional state and asking whether they had any questions about the interview. The interviews were audio recorded and were transcribed verbatim.

R.F. was primarily responsible for analysing the data. This was discussed and explored by their Director of Studies (DoS) who was overseeing the research. The DoS provided considerable support and help with the analysis of the data, the development of themes, and contribution to exploring the relevance of the participant’s narratives. This was in conjunction with J.W.B. who also contributed to discussions about the analysis process. These discussions helped process and develop the analysis.

### 2.5. Data Analysis

The interviews were transcribed manually thus enabling R.F. to become close to the stories [27]. The semi-structured interviews and fieldnotes were initially read as a whole to help develop an understanding of the participant’s experiences of living and working with parental life-limiting illness. This provided an overall context of the collective experiences of the participants. Data was analyzed using Braun and Clarke’s six stages of thematic analysis [28]. This involved reading and becoming familiar with each interview transcription; generating initial codes which related to the aims of the research and then using these to code each transcript, searching the initial codes into potential themes; reviewing and refining the themes, naming the themes and producing the report. Through the stage of repeated reading of the transcripts the emerging ‘big’ themes were identified [29]. These themes were color coded and the relevant chunks of text underscored in the appropriate color. Some of the data slices were relevant to more than one theme and were coded with a number of different colors. All the transcripts were then sorted manually by cutting them up and placing them in coded piles [29]. This process allowed the ‘smaller’ themes to be identified. Throughout this process discussions were regularly held where time was spent reflecting on and exploring the meaning of the emerging themes.

## 3. Ethical Considerations

The study gained ethical approval from the University of Derby, England and the UK National Health Service Research Ethics Committee (07/H1310/23). Informed consent was obtained from all participants and confidentiality and anonymity assured. Permission was sought from the participants to audio record the interviews. Had any observations been made to suggest that a participant was distressed the interview would have been stopped, however, this was not required to be implemented during the fieldwork. Procedures were in place to offer information to participants about support they could access if necessary. No personal data about participants was recorded during the interviews. In the account of the results, the participants have been given assumed names to protect their identities.

## 4. Results

Accounts from children and professionals (Table 1) offered consistent insights into some of the issues children experience when a parent is diagnosed and living with a life-limiting illness or is at the end of life. The major themes to emerge from the study were: communication, emotional and psychological needs, identity, culture, and child development. For the purpose of this paper the communication theme will be the focus. Within this major theme, the smaller themes identified included: parents communicating with children, professionals communicating with children, children communicating with parents, children communicating with professionals, being told what is happening, professional’s anxiety about communicating, awareness of what is happening. The overarching theme was the importance of open, clear and age appropriate communication with children. This was ascertained from the perspectives of the children’s memories of their parent’s illness and the professional’s reflections on their practice. The qualitative methods, where the participants’ voices were paramount, are reflected in the presentation of excerpts to support the themes.

### 4.1. Children’s Perspectives of Communication and Support

Children reflected on their experiences of interactions with their parents throughout the illness. The majority of the children had positive memories about the inclusion; however, their narratives suggested that the quality and quantity of information shared with them was often dependent on their parent’s confidence and ability to talk about the illness. Within their narratives, there needs to be recognition that their age, stage of development and memories of events will have affected their interpretation and recall of what happened.

#### 4.1.1. Quality and Quantity of Information

The quality and quantity of information shared with children was a significant factor for them. Luke reflected on this when he was thinking about the conversations he had been included in with his parents. Luke was nine when his mother was diagnosed with a degenerative neurological disease and fifteen when she died:

“Mum and Dad both talked about the illness and talked to me about it. It felt okay that we had those conversations and I am glad that we did.” (22 years, at age of interview).

Conversations with parents were sometimes remembered as being a positive experience that had been helpful, for the children, in their developing understanding and awareness of what was happening. Through these memories they felt that they had been able to find meaning from the situation. For example, Samantha, whose mother had died from cancer, spoke about how she had been included in conversations with both her parents throughout her mother’s illness:

“It was useful to know what was happening and I would have preferred to know because I didn’t want to be making up stories all of the time.” (17 years).

This was similar to ten-year-old Georgina who reflected about her inclusion in conversations about her father’s cancer:

“I was glad I knew or I would have been more shocked…I wouldn’t like it to be hidden; I knew something was wrong. I would rather have the shock then instead of a big shock at the end.” (10 years).

Younger children too spoke about wanting to know about their parent’s illness. Emma, who was four when her father died recalled:

“Mum told me that Dad was poorly and that the nurses were trying to make him better. She used to ask me if I wanted to know about him.” (6 years).

These reflections show how imperative good communication can be in helping children to begin to manage the situation. However, Samantha also reflected about the potential implications for children if they are not included in conversations about the illness. Samantha’s father worked hard to include her in conversations about the illness, treatments and prognosis:

“We found out a lot from dad because mum was a nurse, but if people don’t know about medicine, they wouldn’t know what’s happening. If people don’t know what is going on they would feel helpless.” (17 years).

The children’s observations show how their experiences and reflections helped shape their understanding of and the importance they placed on communication between children and their parents. Their memories of the illness had been influenced by the quality and quantity of information they have been given.

#### 4.1.2. The Timing of Information

The timing of information and the implications of this was considered by Luke. He talked about the communication and information sharing that he had been involved in with his family and highlighted how this had developed as his mother’s health had deteriorated:

“I suppose in the first couple of years there wasn’t as much to talk about because it was just like a steady progression so it was probably like three years in when you start getting to notice things were actually significantly bad and that’s probably when we spoke about things more.” (22 years).

Luke’s account begins to offer an understanding of the value of incremental, timely information. Although in the early stages of his mother’s illness he was aware of the diagnosis, his recollections would suggest that during this period the family discussions did not focus on the illness. There was a sense during the interview that the conversations developed incrementally as the illness became more invasive and his mother’s health began to deteriorate.

#### 4.1.3. Professional Support

The children’s narratives suggested that when they were included in conversations with their parents about the illness, this inclusion was beneficial. However, their reflections also indicated that there were occasions when there were missed opportunities to talk with the HCPs involved in their parent’s care. For example, Samantha recalled her wish to explore, with a HCP, aspects of her mother’s illness from a professional’s perspective. It was evident that it was important to her to talk to someone who was objective while also knowledgeable about her mother’s case:

“We (Samantha and her 14-year-old sibling; age 11 at time of her mother’s death) were told that we could talk to the nurse but we never got chance because she always visited when we were at school.” (17 years).

Luke also reflected on conversations with his mother’s medical team:

“They did make an effort to explain anything if we asked but I think the main focus was on my mum as opposed to us; which I didn’t mind, I thought that was best because obviously she was the one with the illness.” (22 years).

Sonny, who was five when his father died, found it difficult to remember conversations with the HCPs caring for his father, which was possibly due to his young age at the time of the illness. However, reflecting back on the experience, he said:

“I think it would have been nice to talk to Dad’s doctors ‘cos then I could have asked them stuff about what was wrong.” (9 years).

The quotes provide examples of how children would have liked support from their parents’ HCPs. The timing of HCPs home visits impacted on opportunities for Samantha to talk to professionals about the illness, whereas Luke’s reflections would suggest that he did have some opportunities to talk with his mother’s HCPs. Sonny may have been deemed too young at the time of his father’s death to be included in conversations. However, upon reflection he too thought that, even at a young age, it would have been helpful to have the opportunity.

In contrast, Jennifer’s story was very different. Jennifer had been a young carer for her mother for over ten years. Her mother was diagnosed with a degenerative neurological disease when Jennifer was three years old. She lived with her mother and older sister. The illness meant that her mother had periods of hospitalisation which became the norm for Jennifer. However, throughout this period, she could not recall being given any information about her mother’s illness or ‘constructive professional support’. A substantial part of her life and biography was about living with and caring for an ill mother; however, Jennifer did not know that her mother would die from the illness. She reflected about the anger and hurt that she felt following her mother’s death. A factor being that no HCPs or other professionals involved in her mother’s care had spoken to her about the impending death:

“I think I do vaguely remember like the odd nurse but not really that much. I don’t really remember much medical attention it was just like sorting out medication and that was it nobody ever talked to me about mum’s illness of what was going to happen to her.” (24 years).

She felt that there had been a ‘conspiracy of silence’ and that as a result she had not had the opportunity to prepare for the death. She felt that she had been let down by professionals who had not, in her opinion, taken her needs into account.

### 4.2. Professionals’ Perspectives

This section focuses on the professionals’ perspectives about communication with parents and children and their clinical roles. The professionals interviewed had direct experience of working in palliative care, with children and families. As a result, they had strong opinions about what might constitute good practice and recognized the importance of providing holistic care and support.

#### 4.2.1. Appropriate Adults to Have Conversations with Children

During the interviews, professionals considered who, in their opinion, were the most appropriate people to talk to children about parental illness. A clinical nurse specialist reflected about her role in supporting parents to have conversations with their children:

“So a lot of the work that I do is helping parents with those kinds of conversations because I do feel that parents are actually the people who are best placed and best suited to do that work but it is scary stuff.”

The theme of parents being the most appropriate people to talk to their children about the illness was also highlighted by a bereavement co-ordinator in a hospice:

“…what we try and promote is really simple, we give people adequate information because how we work is a child can be told anything, anything, it just has to be told by people who know him or her.”

However, this view was in contrast to some of the accounts from children where they also wanted to speak with HCPs who, in the children’s opinion, could provide them with a more objective perspective. The professionals’ experiences of direct work with children and families had been influential in developing their knowledge about a preferred way of working. Through their practice they recognized the value of supporting parents to engage in the ‘scary stuff’ of talking to children about the illness. However, there was recognition and acknowledgment that this work can be difficult, especially if the professional does not routinely work with children and families.

#### 4.2.2. Professional Fear

While the professionals felt that it was good practice to support parents to engage in conversations with their children about the illness, they also spoke about how they often observed a ‘professional fear’ from colleagues who do not work specifically with children. This professional fear was often underpinned with fear of communicating with children:

“We find a lot of people and services aren’t really (emphasized) available for children because either through fear of getting it wrong or they think they are going to make it worse when the worst has already happened, the diagnosis has been given, the bombshell has been dropped and the person has died or the person is dying so there is nothing we can do to make it worse.”

The theme of there being a ‘professional fear’ when talking to children about their parent’s illness was also described by a social worker employed in a hospice:

“Some people are very honest and they will admit that they are terrified of saying the wrong thing.”

A nurse practitioner discussed the ‘professional fear’ from a different perspective. She recognized the need to access training and to develop her skills, but also knew that institutional barriers would prevent this:

I think really such as myself needs more training to be able to deal with children. I mean you know if I went and said to my managers “I need some training on child bereavement issues”, they would say “what for you are an adult service”. You know and I would say “well you know I’ve got relatives whose kids need help” and they would say “well refer them to social workers, let the teachers know, refer them to the children’s community Macmillan nurse”.

The nurse’s experience provides an example of how limited training opportunities could affect practice and as a result add to ‘professional fear’ of working with, communicating with and supporting the children of patients. The observations from the professionals provide an indication of some of the difficulties colleagues experience if they have not received training to work with children and families. However, a general consensus was that part of the HCP’s role is to empower parents so that they begin to have more confidence to talk with their children about the illness.

#### 4.2.3. Barriers to Communication

The professionals shared their frustrations of not always being able to provide the support families wanted, in addition to their observations of colleagues who do not engage with families. They talked about some of the barriers that prevented them from being more involved. Some frustrations were rooted in professional practice. These included not having time within workloads, not having training in this area and therefore feeling ill-equipped to begin any meaningful conversations, and not feeling confident to support parents or children. A clinical nurse specialist gave an example of this from their observations of colleagues:

“So many of the things are around these conversations taking too much time, you know if you open it up you will be opening a can of worms and that you won’t have the skills or you won’t have the emotional reserves to be able to manage it.”

During the interviews there was evidence of a distinction between professionals who regularly work with children and families and those who don’t. Not surprisingly professionals who, within their day to day role, are involved in supporting parents and their children were more confident and had a greater understanding of the importance of providing support for children, whether through direct work or indirectly by supporting the children’s parents.

## 5. Discussion

**Figure 1:**
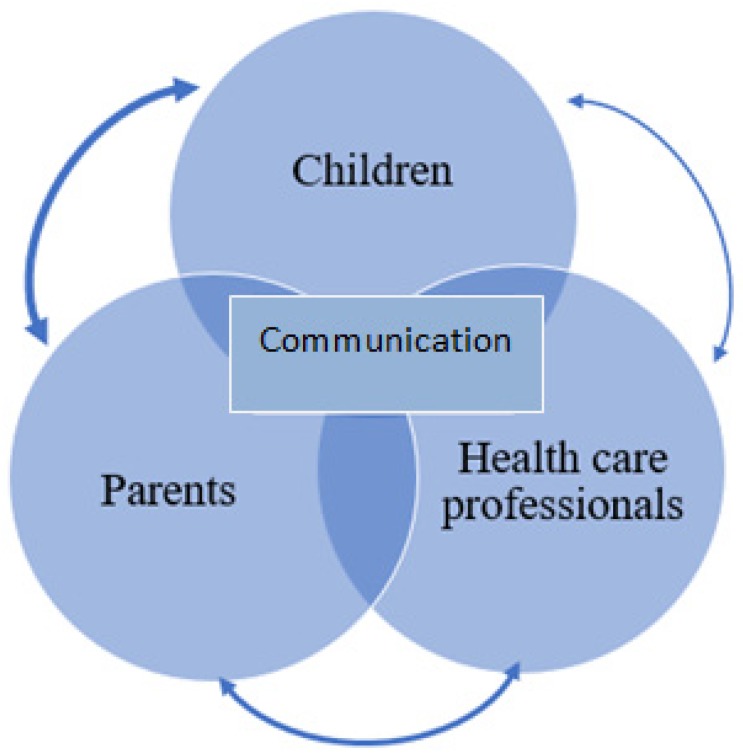
An illustration of the bi-directional communication interrelationships between children, parents and HCPs. For children to be informed, in an age appropriate way that also considers their emotional needs, about their parent’s illness, they need information to be communicated with them from their parent(s) and sometimes also directly from HCPs. Within this interrelationship, HCPs also have an important role in assessing, evaluating and understanding the information needs of parents and their children, to help empower parents to effectively communicate with their children. The weighted lines represent the likely importance of the different interrelationships, although these will differ for each family. For example, HCPs often help support and enhance communication between parents and children but are sometimes involved in direct communication with children.

The complex inter-relationship of communication between parents, who have a life-limiting illness, their children and professionals involved in their care has been explored (Figure 1). From the children’s perspectives, it was evident that they all knew that their parent was ill but did not always know the extent of the illness and prognosis. The quality and quantity of information about the illness was important for how they remembered the illness period and importantly how they managed their distress during this time.

Children reflected on their experiences of interactions with adults during their parent’s illness. Here there was a distinction between interactions with familiar adults, for example parents and the professionals who were caring for their parent. The majority of children recalled that their parents did speak to them about the illness and that this had been helpful. Conversely their recollections of interactions with professionals tended to be less positive. This was sometimes attributed to structural barriers, for example the nurse visiting the home during the day when they were at school. It would therefore be helpful if practice structures were in place whereby children could access their parent’s HCPs. This would give them the opportunity to talk about the diagnosis, treatments, and prognosis with an objective person who could provide factual answers. This is consistent with previous work which has evidenced how bereaved children engage positively in opportunities to talk with medical doctors about illness and death [30]. In these circumstances they look to the doctor for information, which helps them write their ‘last chapter’ about the person who has died [30]. Both pre- and post-death, children are actively looking for information to help them make sense of what is happening.

The value of communication and information sharing was seen as an important requirement in enabling children to make some sense of what was happening within their family. The risk that, without adequate information, children will ‘make up stories’ was analyzed as a significant observation. When given little or no information they will create ‘information’ through fantasy and conjecture [11]. The risk being that the ‘made up’ version is likely to be inaccurate and could potentially increase their anxieties [8]. Samantha had clearly thought about this and had understood the implications of not being told, in addition to recognizing that being told is better than the alternative of not knowing. This highlights the importance of not only talking but also listening to children and engaging in discussions that explore their understanding of what they think is happening.

The narratives also indicated that a lack of information can affect children’s understanding of what is happening, in part because they are dependent on adults to teach them what they do not know [11]. Information about the illness is recognized as being important in helping children develop a ‘durable biography’ [31] and helps in the writing of their ‘penultimate chapter’ [32]. This is a prospective chapter that is ‘written’, by children, during the parent’s illness. The quality of its content is dependent on the extent to which children are included in conversations and information sharing about the illness [32]. However, it must be recognized that developmental difference, based on age, is an important factor that will affect how children process information about the illness. This needs to be taken into consideration when conversations between children and adults are taking place.

Parents often look to their HCPs for guidance about how to talk with their children about their diagnosis, but there is often a discrepancy between the support parents would like and what is offered [9,33,34]. The value of parents talking to their children about the illness was recognized by the professionals involved in this research. They believed that children would have the capacity to cope with information if they were given it by familiar adults. Previous research supports this [35], however it also suggests that while parents are generally the most appropriate people to communicate the news to their children, they often need considerable guidance and help from professionals to know how to start such difficult conversations [35]. For example, mothers diagnosed with cancer had concerns that their children’s needs were not included within the professional support they were offered [36]. A systematic review [14] highlighted the discrepancy between what parents with a life-limiting illness want from their HCPs, with regards to talking to their children about the illness, and what they receive. The paper showed that parents want and need information and guidance from their HCPs at the point of diagnosis and throughout the illness; however, this support is rarely offered or provided. This is contrary to the current study where the professionals recognized the importance of talking to children about the illness. However, it should be noted, that the professionals involved in the current research, all worked regularly with children and families and therefore had practical experience, knowledge and confidence to undertake this ‘scary stuff’. This highlights how important it is for HCPs and other professionals to be available for children and to feel confident to take time to listen and offer support.

The professional’s narratives showed a clear distinction between clinicians who within their palliative care specialism work regularly with children and families, and those who focus specifically on the ill patient. A tension is that HCPs often do not feel confident to engage in conversations that involve the sensitive subject of children and their needs [9,33]. An overarching barrier that was identified was the ‘professional fear’ that is held by HCPs. Within this fear are concerns about ‘getting it wrong’ or making the situation worse by talking about it. This is congruent with findings from a recent systematic review [14] that HCPs are often reluctant to address these concerns because of fears of insufficient time and expertise. However, the current study did not explore whether HCPs empowered parents to have conversations with their children. It is therefore impossible to ascertain whether the children involved always knew the extent of involvement from their parent’s HCPs. However, what is evident is that if more robust systems were in place that addressed the needs of children whose parents have a life-limiting illness, there may be scope for more support to be offered. This could include consideration of collaborative work across professions and institutions whereby HCPs refer, when appropriate, children and their families to specialist provision for support. However, for this to occur there remains the need for professionals, from different care providers, to be able to access training to meet the needs of this community of potentially vulnerable children.

## 6. Limitations

This study has several limitations. The professionals interviewed were closely involved in palliative care services and all had involvement with children and families as part of their role. Therefore they were able to reflect on direct work with children and families and moreover, were confident in their practice. There was no involvement of HCPs who work solely with patients and who do not work with or support the patient’s children. Therefore, their perspectives have not been represented.

Methodological limitations include that the age range of children was extremely broad and moreover, the length of time since the bereavement was also wide ranging. Both of these factors are likely to have had a significant impact on their memories of the period when their parent was ill. This could impact on their responses within the study. In addition the sample size for the children might limit the variety of narratives available for interpretation. Due to difficulty obtaining access to and recruiting children prospectively during the recruitment stage of the research, the initial aim of interviewing children who were living with an ill parent needed to be changed to a retrospective sample. This therefore has the potential for recall bias which could affect how the children remember and report events. This might have been different to how they were at the time and how the children might have reported them contemporaneously. This remembered perspective is, however, important as it is what the children are living with. To accommodate the recruitment changes there was also then inclusion of professionals who worked within palliative care or allied professions. It was intended that this broad range of professionals would provide different perspectives about working with children and their families. A further limitation was that the voice of surviving parents, who would have had an important perspective, was not included in the research.

## 7. Implications for Practice

The participant’s narratives have highlighted the complexity of providing holistic support to all the family when a parent has a life-limiting illness. An implication for practice is how the distinct needs of the parent, their children and the professionals involved in their care can be most effectively combined. However, it was evident that central to this is the value and importance of communication. The paper has highlighted a distinction in practice between professionals who routinely work with children and families, when a parent has a life-limiting illness, and those who do not. The HCPs involved in this research were often involved in work with children and their families and therefore generally felt more comfortable talking with and supporting families. However, they also reflected on observations of colleagues and practitioners who do not routinely engage with families. Their observations were that this lack of contact could result in there being a ‘professional fear’ preventing conversations from being undertaken and support being offered. This observation could have serious implications for practice as it could be a significant reason why some families do not get the best available support. An additional factor that might result in some HCPs having a ‘professional fear’ is the limited training available in this area of practice. If training in working with, and supporting children and families during parental life-limiting illness became mandatory, all professionals who come into contact with them would, as a minimum, have a better understanding of some of the issues faced and how best to provide support [17].

Samantha highlighted an important implication for practice when she recalled the desire to speak with her mother’s nurse about the illness. This did not happen because of the timing of home visits by the nurse. Therefore, if systems could be in place which offered some flexibility around home visits, or that provided time and space for children to meet with their parent’s HCPs this could provide an important opportunity for children to find out more about what is happening. Two important issues are highlighted in this refection from Samantha. Firstly, there needs to be more research to develop our understanding of the extent to which children want to talk with their parent’s HCPs. Within this, we also need to learn more about what these conversations would ideally be about and the timing of them. Secondly more work is required to explore how structural barriers can be most effectively managed so that children can meet with the HCPs.

The findings add to the growing research that children want to be involved in conversations and information sharing about their parent’s illness. However, parents often find it difficult to know what is best in terms of talking to their children about the diagnosis and prognosis. In these situations they often look to their HCPs for support and guidance about how to talk with and support their children. However, as the paper has highlighted, unless HCPs are trained and work regularly with families they often feel unable to offer support. Therefore, a recommendation for practice is that training in supporting parents with a life-limiting illness, would be beneficial to equip HCPs with the necessary skills, knowledge and confidence to help support parents to talk with their children.

## 8. Future Research

Future research to build on this work, and the work of others, to add to our theoretical and practical knowledge of the needs of children when a parent has a life-limiting illness is needed. A longitudinal study interviewing the triad of parents, their children and HCPs involved in their care, at intervals throughout the illness and into bereavement would provide ongoing insights from different perspectives. In addition, an in-depth study focussing on HCP’s reflections of working with parents and children would be helpful to develop our understanding, from their perspective, of the challenges and opportunities of this work. There is scope for explorations about how positive inter-agency work could be accomplished so that HCPs can best work with other professionals involved with the family, for example schools and education services, so that a more holistic package of care can be provided.

## 9. Conclusions

Parents upon receiving a life-limiting diagnosis often struggle to know how, what or when to tell their children about their illness [1,8,9,10,37,38]. Typically they look to their HCPs for guidance, however HCPs are often unable, or don’t provide this support [14]. Prominent factors contributing to this include there being a ‘professional fear’ about talking with children, not having time to begin conversations and institutional boundaries whereby adult and children’s services are distinct. The current research supports existing accounts about the importance and relevance of communication between HCPs, parents and children. There is an interrelationship whereby children want and need to have conversations with their parents (or other trusted adults) about the illness and similarly parents look to their HCPs for support in doing this.

Conversations about dying and death can be difficult to manage, but when parents are not supported, by HCPs, to talk to their children about their illness there is a risk that opportunities for supportive work are missed. These missed opportunities are likely to impact on all family members, but children are most at risk of being excluded from information about what is happening in their family. The findings suggest that HCPs need to be empowered to support parents and children. When they do not routinely work with the patients’ children they do not always have the confidence to offer support because of ‘professional fear’. However, if this fear was alleviated through training this would provide a level of empowerment that would benefit parents, children and the HCPs.

## Figures and Tables

**Table 1:** Research Participants included in this study, divided into children and professions, with key demographics.

Children, Gender and Age when Bereaved	Age When Interviewed	Parent’s Illness/Gender
Jennifer, female, 15 years	24 years	Multiple Sclerosis/Mother
Luke, male, 15 years	22 years	Motor Neurone Disease/Mother
Georgina, female, 8 years	10 years	Bowel Cancer/Father
Samantha, female, 15 years	17 years	Breast Cancer/Mother
Kirsti, female, 9 years	11 years	Lung Cancer/Father
Emma, female, 4 years	6 years	Lung Cancer/Father
Sonny, male, 5 years	9 years	Motor Neurone Disease/Father
**Professionals**	**Profession**	**Place of Work**
Hannah	Nurse Practitioner	Hospital
Dr. Jones	Counsellor	Independent Organisation
Elizabeth	Palliative Care Social Worker	Primary Care Trust
Christopher	Social Worker	Hospice
Pat	Service Manager	Voluntary Organisation
Harriet	Palliative Care Nurse	Hospital
Carol	Counsellor	Hospice
John	Bereavement Co-ordinator	Hospice
Susan	Social Worker	Hospice
Amanda	Education Welfare Officer	School
Charlotte	Social Worker	Hospice
Martha	Social Worker	Hospice
Anne	Palliative Care Nurse	Hospital
Alison	Play Therapist	Voluntary Organisation
Julia	Bereavement Support Worker	Voluntary Organisation
Catherine	Community Palliative Nurse	Hospice

All the names used are pseudonyms.
